# Dynamic properties of the *Sulfolobus* CRISPR/Cas and CRISPR/Cmr systems when challenged with vector-borne viral and plasmid genes and protospacers

**DOI:** 10.1111/j.1365-2958.2010.07452.x

**Published:** 2011-01

**Authors:** Soley Gudbergsdottir, Ling Deng, Zhengjun Chen, Jaide V K Jensen, Linda R Jensen, Qunxin She, Roger A Garrett

**Affiliations:** Archaea Centre, Department of Biology, University of CopenhagenOle Maaløes Vej 5, 2200N Copenhagen, Denmark

## Abstract

The adaptive immune CRISPR/Cas and CRISPR/Cmr systems of the crenarchaeal thermoacidophile *Sulfolobus* were challenged by a variety of viral and plasmid genes, and protospacers preceded by different dinucleotide motifs. The genes and protospacers were constructed to carry sequences matching individual spacers of CRISPR loci, and a range of mismatches were introduced. Constructs were cloned into vectors carrying *pyrE/pyrF* genes and transformed into uracil auxotrophic hosts derived from *Sulfolobus solfataricus* P2 or *Sulfolobus islandicus* REY15A. Most constructs, including those carrying different protospacer mismatches, yielded few viable transformants. These were shown to carry either partial deletions of CRISPR loci, covering a broad spectrum of sizes and including the matching spacer, or deletions of whole CRISPR/Cas modules. The deletions occurred independently of whether genes or protospacers were transcribed. For family I CRISPR loci, the presence of the protospacer CC motif was shown to be important for the occurrence of deletions. The results are consistent with a low level of random dynamic recombination occurring spontaneously, either inter-genomically or intra-genomically, at the repeat regions of *Sulfolobus* CRISPR loci. Moreover, the relatively high incidence of single-spacer deletions observed for *S. islandicus* suggests that an additional more directed mechanism operates in this organism.

## Introduction

CRISPR adaptive immune systems of archaea and bacteria consist of clusters of identical repeats separated by unique spacer sequences of constant length adjoining a low complexity leader sequence carrying consensus sequence motifs, and physically linked to groups of *cas* genes (Jansen *et al*., 2002; Tang *et al*., 2002). Less frequently they are linked, at least functionally, with cassettes of *cmr* genes (Hale *et al*., 2009). They occur in the sequenced chromosomes of almost all archaea and about 40% of bacteria, as well as in some plasmids (reviewed in Lillestøl *et al*., 2006; Makarova *et al*., 2006; Grissa *et al*., 2008; Karginov and Hannon, 2010). The spacer regions constitute functionally active units which derive from extrachromosomal DNA of viruses and plasmids (Bolotin *et al*., 2005; Mojica *et al*., 2005; Pourcel *et al*., 2005; Lillestøl *et al*., 2006). For crenarchaea many spacers have been shown to match viral genomes or plasmids, despite the former often coexisting in a stable relationship with their hosts (Prangishvili *et al*., 2006a; Lillestøl *et al*., 2009; Shah *et al*., 2009). Whole CRISPR loci are transcribed from promoters within the leader regions and transcripts are then processed to yield smallest products in the range 35–45 nt containing most of the spacer sequence (Tang *et al*., 2002; 2005; Lillestøl *et al*., 2006; 2009; Brouns *et al*., 2008; Hale *et al*., 2009). Transcript processing is executed specifically within the repeat regions by Cas or Cmr proteins and the small crRNAs corresponding to the spacer and an adjoining part of the repeat are then transported to the invading genetic element (Brouns *et al*., 2008; Hale *et al*., 2008; 2009;). crRNAs, of different sizes, target either DNA (Marraffini and Sontheimer, 2008; Shah *et al*., 2009) or RNA (Hale *et al*., 2009) facilitated by groups of Cas and Cmr proteins respectively. Annealing of a crRNA–Cas protein complex to a complementary protospacer DNA sequence is presumed to facilitate inactivation, probably via degradation of the genetic element (Marraffini and Sontheimer, 2008; Shah *et al*., 2009).

Genome comparisons have indicated that new spacer-repeat units are inserted at the junction of the leader region and the first repeat for both archaea and bacteria (Pourcel *et al*., 2005; Lillestøl *et al*., 2006; 2009;) and this has been demonstrated experimentally for the bacterium *Streptococcus thermophilus* where a new spacer deriving from a phage genome was inserted into a CRISPR locus in response to phage infection, which in turn led to phage resistance (Barrangou *et al*., 2007). Moreover, it was demonstrated for this bacterium that perfectly matching spacer and protospacer sequences are essential to produce effective immunity via the CRISPR/Cas system (Barrangou *et al*., 2007; Deveau *et al*., 2008; Horvath *et al*., 2008). Thus, repeatedly invading genetic elements lead, potentially, to the addition of new spacer-repeat units at the leader end of a CRISPR locus and to a continual increase in its length. Many archaeal CRISPR loci carry 100 or more spacer-repeat units, but mechanisms exist to limit their sizes (Lillestøl *et al*., 2006). Although no direct evidence has been obtained for systematic fragmentation and subsequent loss of CRISPR loci fragments, a comparison of partially overlapping CRISPR loci of *Sulfolobus solfataricus* strains P2, P1 and 98/2 revealed that large indels, probably deletions, had occurred within some loci. Furthermore, two apparently inactive CRISPR loci E and F were found that were identical in sequence in strains P1 and P2, and the latter was deleted in strain 98/2, suggesting that loss or inactivation of linked Cas genes or mutation of the leader region had occurred (Lillestøl *et al*., 2006; 2009; Shah and Garrett, 2010).

Here we investigate the fidelity of crRNA–protospacer interactions for the CRISPR/Cas system of the crenarchaeal thermoacidophile *Sulfolobus*, and the stability of the CRISPR/Cas modules, when challenged by recently developed vector constructs (Deng *et al*., 2009) carrying viral or plasmid genes or protospacers, maintained under selection, which show varying degrees of sequence complementarity to host CRISPR spacers.

## Results

### Viral genes carrying spacer matches under selection can produce CRISPR loci deletions

**Fig. 1:**
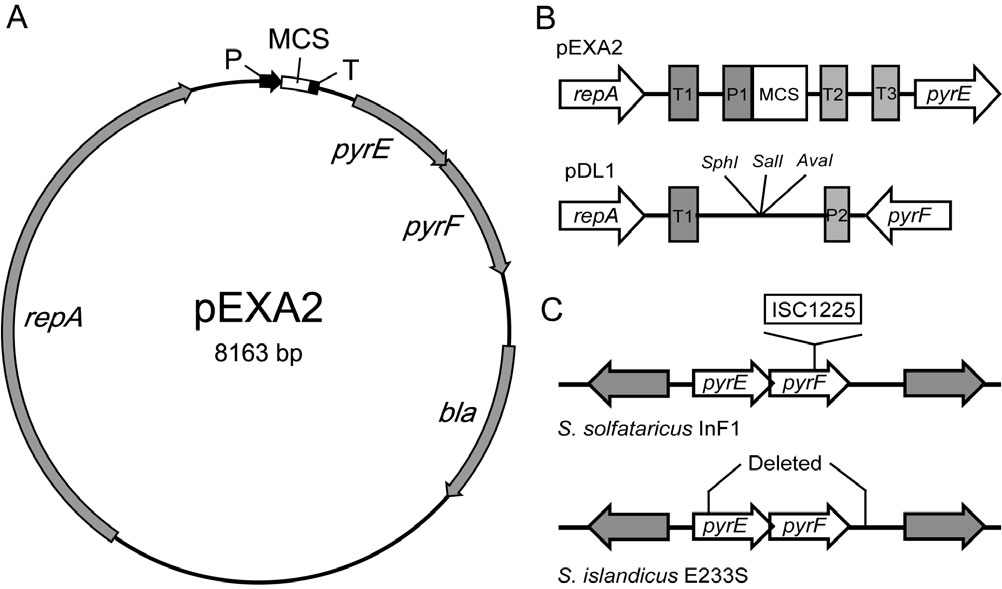
Scheme showing the vector–host constructs. A. pEXA2 was produced by modifying the *Sulfolobus*–*E. coli* shuttle vector pZC1 (Peng *et al*., 2009). B. The cloning region of the two vectors showing positions of promoters P1 and P2, where P1 is arabinose-inducible, the terminators T1 to T3, and cloning sites for viral genes and protospacers including a multiple cloning site (MCS) in pEXA2. C. Mutations in the *pyrE/pyrF* genes of the uracil auxotrophic hosts *S. solfataricus* LnF1 (Z. Chen *et al.*, unpublished) and *S. islandicus* E233S (Deng *et al*., 2009) are indicated.

**Fig. 2:**
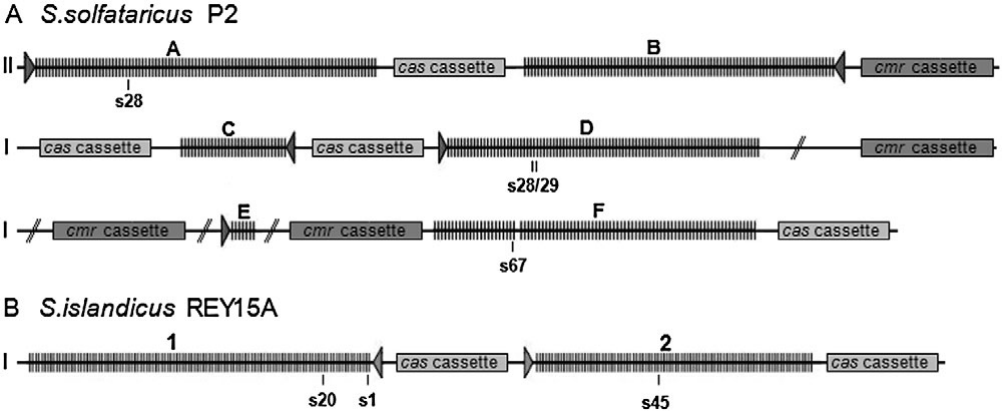
Schematic representation of (A) the six CRISPR loci A to F of *S. solfataricus* P2 and (B) CRISPR loci 1 and 2 of *S. islandicus* REY15A, drawn to scale (extending from genomic positions 725 000 to 742 500), together with the cassettes of *cas* genes and *cmr* genes. CRISPR loci A to D (genomic positions 1 233 400–1 311 600) and CRISPR loci E and F (positions 1 744 000–1 815 500) are clustered within the *S. solfataricus* P2 genome. The positions of spacers matching protospacers within the vector constructs are indicated where ‘s’ denotes spacer followed by the number of the spacer measured from the leader region, indicated by an arrowhead. Sulfolobales family types of CRISPR/Cas modules are indicated by I and II.

Initial experiments were performed on the auxotrophic mutant *S. solfataricus* InF1, carrying an inactivated *pyrF* gene and using the expression vector pEXA2 modified from a *Sulfolobus*–*Escherichia coli* shuttle vector pZC1 (Peng *et al*., 2009) and containing an arabinose-inducible araS promoter (P_1_) as illustrated (Fig. 1). Three genes from the *Acidianus* two-tailed bicaudavirus (ATV) (Prangishvili *et al*., 2006b) were cloned into the multiple cloning site and were modified to generate perfect matches to single spacers in CRISPR loci A or D of *S. solfataricus* P2 (Fig. 2A). The genes correspond to: ATV618 encoding an AAA+ ATPase matching spacer 29 of CRISPR locus D; ATV145 encoding a putative DNA-binding protein matching spacer 28 of CRISPR locus D, and ATV892 encoding a Von Willebrand Factor A domain protein matching spacer 28 in CRISPR locus A. The protospacers of these viral genes showed perfect sequence matches to the CRISPR spacers and conserved CC or CT Protospacer Associated Motifs (PAM) located 5′ to the DNA strand, corresponding to the crRNA transcribed from spacers of family I and II CRISPR loci D and A, respectively, were also present (Lillestøl *et al*., 2009; Shah *et al*., 2009). Whereas the mRNAs of ATV618 and ATV892 carried protospacers complementary to crRNAs from the matching spacers, the crRNA matched the reverse transcript for ATV145.

ATV618 and ATV892 expression resulted in a slightly slower growth rate but after 50 generations mRNAs were still expressed and the vector was unchanged. ATV145 production led to slower cell growth, indicative of some toxicity, and little mRNA was expressed after 50 generations. Analysis of the vector revealed that mutations had occurred including an IS element insertion into the arabinose-regulatory site upstream from the promoter, thereby reducing transcription, and point mutations within the promoter and ORF but no mutations were observed in the protospacer or its adjacent PAM motif (data not shown).

**Fig. 3:**
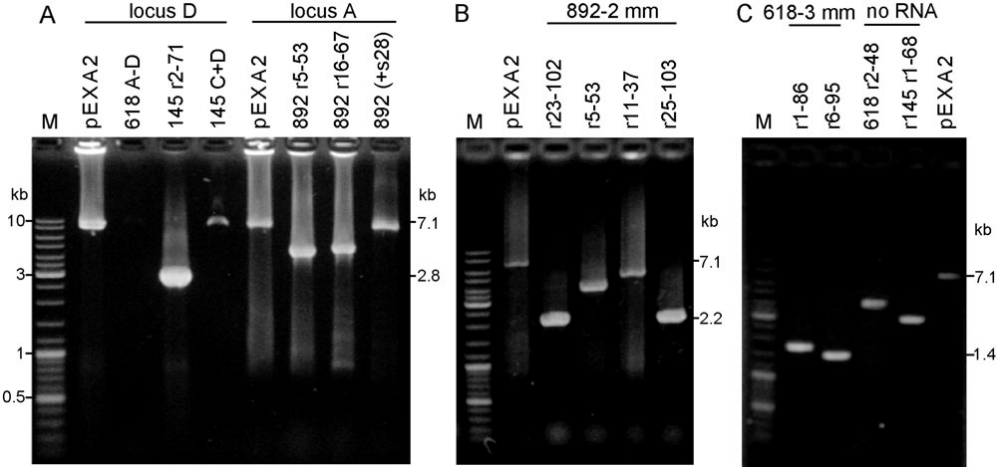
Agarose gels showing examples of PCR products obtained from CRISPR loci of transformants of *S. solfataricus* P2-InF1 carrying genes for ATV618, ATV145 and ATV892 cloned into pEXA2. A. PCR products obtained from CRISPR loci after challenging with constructs carrying perfect matches to spacers in locus D (ATV618 and ATV145) and locus A (ATV892). The bracketed spacer number denotes the presence of the matching spacer 28. B. PCR products amplified from locus A of transformants with ATV892 carrying two mismatches in the protospacer sequence. C. PCR products from locus D when challenged by ATV618 containing three mismatches in the protospacer, and two samples showing perfect matching protospacers with no transcription. M indicates DNA size markers. The illustrated PCR products yielded the results for estimating the deletion sizes marked with asterisks in Table 1.

Although transformation efficiencies could only be estimated approximately (see *Experimental procedures*), each construct yielded very few viable transformants. These were grown in liquid medium lacking uracil and the DNA was examined first by PCR for the presence and size of the CRISPR loci A or D which carried the matching spacer. The results revealed that most viable transformants carried a major deletion. Examples of PCR products derived from the deletion mutants are presented in Fig. 3A and all of the results are summarized in Table 1 where the estimated sizes of the deletions are given. For ATV618, the CRISPR/Cas modules A to D were absent while for ATV145, CRISPR/Cas modules A to D or C to D (Fig. 2) were absent from different transformants. In addition, large internal deletions, which included the matching spacer, were observed within CRISPR locus D for ATV145 and locus A for ATV892 (Fig. 3A; Table 1). We conclude that challenging the CRISPR systems with viral genes which are under selection, and carry perfectly matching protospacers with family-specific PAM motifs, yields primarily transformants carrying each specific spacer deletion which can grow under selection.

**Table 1:** Challenging the CRISPR systems of *S. solfataricus* P2 with modified bicaudavirus ATV gene inserts carrying matching protospacers and corresponding PAM motifs inserted into pEXA2 (Fig. 1).

					CRISPR loci deletions	
					
ATV gene insert	PAM motif	CRISPR locus/spacer	Transcript complementary to crRNA	Protospacer mismatches (position)	PCR estimate (kb)	Sequencing result
ATV618	CC	D/29	mRNA	0	> 90*	Loci A,B,C,D*
ATV618	CC	D/29	None	0	3.3	r5–r46
					3.4*	r2–r48*
ATV618	CC	D/29	mRNA	1 (24)	> 90 (×2)	Loci A,B,C,D (×2)
					> 30	Loci C,D
					1.5	r10–r31
					0 (×2)	Spacer present (×2)
ATV618	CC	D/29	mRNA	2 (18, 24)	0 (×2)	Spacer present (×2)
ATV618	CC	D/29	mRNA	3 (18, 21, 24)	5.4*	r1–r86*
					6.1*	r6–r95*
ATV145	CC	D/28	Reverse	0	> 90	Loci A,B,C,D
					> 30*	Loci C,D*
					4.4*	r2–r71*
					3.5	r6–r59
ATV145	CC	D/28	None	0	4.4*	r1–r68*
					4.6	r2–r73
ATV892	TC	A/28	mRNA	0	3*(×2)	r5–r53*(×2)
					3.3*	r16–r67*
					0*	Spacer present*
ATV892	TC	A/28	mRNA	2 (10, 19)	4.9*	r23–r102*
					4.8*	r25–r103*
					3.1*	r5–r53*
					1.5*	r11–r37*
pNOB8-315	CG	F/67	mRNA	0	0 (×2)	Spacer present (×2)
pNOB8-315	CC	F/67	mRNA	0	0 (×2)	Spacer present (×2)

Deletions in CRISPR loci, and of whole CRISPR/Cas modules, were estimated from the sizes of PCR products and the positions of the CRISPR loci deletions were determined accurately by sequencing. All internal CRISPR locus deletions showed normal spacer–repeat junctions. Exceptionally for the ATV145 gene, the protospacer is inverted such that the reverse transcript is complementary to the crRNA. PCR products shown in Fig. 3 provide a basis for estimating deletion sizes that are indicated by asterisks in columns 6 and 7. r5–r46 denotes that recombination has occurred between repeats 5 and 46 resulting in the loss of the intervening spacers and repeats. Loci A,B,C,D means that the whole region carrying the loci and their associated *cas* genes has been deleted. (×2) shows that the result was obtained independently twice. Approximate transformation efficiencies for the viral gene constructs (see *Experimental procedures*) were in the range 0.1–2.0% of the pEXA2 control [5.3 × 10^4^ cfu (µg DNA)^−1^] and for the pNOB-315 gene constructs they were in the range 7 × 10^3^ to 2.5 × 10^4^ cfu (µg DNA)^−1^.

**Fig. 4:**
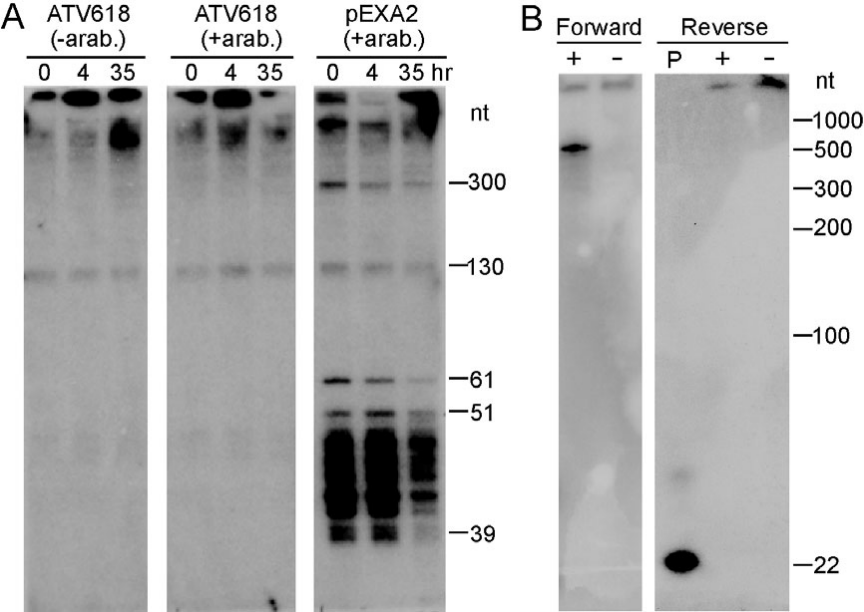
A. Northern blot probing for crRNAs transcribed from spacer 29 of locus D of *S. solfataricus* P2. The transformant which apparently lacks CRISPR loci A to D (Table 1) was probed. The vector construct carried the ATV618 gene with a matching protospacer and CC motif. Samples were tested without and with arabinose stimulation of ATV618 mRNA transcription to test whether there was any effect of the mRNA. pEXA2 denotes transformants carrying the empty vector with no insert. Normally processed crRNAs are only visible for transformants carrying the empty vector. B. Northern blot probing for the protospacer region of ATV145 transcripts from forward and reverse strands. + denotes the total RNA isolated from the pEXA2-ATV145 transformant and − indicates the total RNA isolated from the transformant of the construct pEXA2-ATV145 for which the *araS* promoter had been exchanged with an archaeal transcriptional terminator (see main text). ‘P’ represents a positive control where a 10 pmol 22-nt-long DNA oligonucleotide is employed that is complementary to the probe. Sequences of oligonucleotide probes are given in *Experimental procedures*.

Northern blotting experiments were performed to test for the presence of crRNAs produced by the host cells. An example is shown (Fig. 4A) for cells transformed with the vector carrying the ATV618 gene which produced a large deletion, encompassing CRISPR loci A, B, C and D (Fig. 3A; Table 1). The total cellular RNA extract was probed with an oligonucleotide complementary to the crRNA of the matching spacer 29 of locus D (Fig. 2). In the presence or absence of arabinose, no crRNA was detected for spacer 29 of transformant samples harvested at different times, whereas a typical range of processed products, including the smallest crRNAs (Lillestøl *et al*., 2006; 2009;), were observed in the control sample carrying empty pEXA2 (Fig. 4A).

The effects of ATV618 gene inserts carrying single-, double- and triple-spacer mismatches were also examined and vector constructs were made with base-pair changes within the protospacer region of the gene as indicated (Table 2). For one, two or three base-pair mismatches again a low number of viable transformants was obtained and they were analysed for deletions in the CRISPR loci. For the single mismatch, six transformants were analysed four of which showed major deletions (Table 1). Two lacked the region carrying CRISPR/Cas modules A to D, another lacked modules C and D, and a fourth carried a large internal deletion in locus D (r10 to r31) including matching spacer 29. No changes in CRISPR locus D were detected for the remaining two transformants (Table 1). The construct with two mismatches yielded only two transformants which produced PCR products of unaltered size from locus D each with an intact spacer 29 and the host genome changes were not identified. In a parallel experiment with two mismatches introduced into the ATV892 protospacer (Table 2), four transformants were obtained. PCR products obtained across the deletions, illustrated in Fig. 3B, were analysed and for each one large deletions within locus A were identified (Table 1). Three mismatches in the ATV618 protospacer yielded two transformants showing different large deletions (r1–r86, r6–r95) including spacer 29 (Fig. 3C; Table 1).

**Table 2:** DNA strands of cloned protospacers showing the sequence corresponding to crRNAs.

Construct	Locus/spacer	Sequence	Mismatches	Positions
(A) *S. solfataricus* P2				
ATV618	D/29	**CC**ATTTTGATAACTAGATGTGGAACCGAAGTTTACTACTAGTT	0	
	D/29	**CC**ATTTTGATAACTAGATGTGGAACC**A**AAGTTTACTACTAGTT	1	24
	D/29	**CC**ATTTTGATAACTAGATGT**A**GAACC**A**AAGTTTACTACTAGTT	2	18, 24
	D/29	**CC**ATTTTGATAACTAGATGT**A**GA**G**CC**A**AAGTTTACTACTAGTT	3	18, 21, 24
ATV892	A/28	**TC**CCTCGCTAACGTTCAAATCTTTCAATAATTTTTGCACGT	0	
	A/28	**TC**CCTCGCTAAC**A**TTCAAATC**C**TTCAATAATTTTTGCACGT	2	10, 19
ATV145	D/28	**CC**GAAAAGCCAATCCCAAGATACATCATCGCAGAAATATTCA	0	
Protospacer	A/28	**TC**CCTCGCTAACGTTCAAATCTTTCAATAATTTTTGCACGT	0	
	A/28	**TC**C**T**TCGCTAACGTTCAAATCTTTCAATAATTTTTGCACGT	1	1
	A/28	**TC**CCTCGCTAACGTTCAAATC**C**TTCAATAATTTTTGCACGT	1	19
	A/28	**TC**CCTCGCTAACGTTCAAATCTTTCAATAATTTTTGCACG**C**	1	38
	A/28	**TC**C**T**TCGCTAACGTTCAAATCTTTCAATAATTTTTGCACG**C**	2	1, 38
	A/28	**TC**CCTCGCTAACGTTCAAATCTTTCAATAATTTTTGC**GGCC**	4	35–38
	A/28	**TC**CCTCGCTAACGTTCAAATCTTTCAATAAT**CGGCCGCACA**	10	29–38
(B) *S. islandicus* REY15A				
Protospacer	2/45	**CC**ATTAGGAGTCGTAGCACAGGGAGCTGTACAGTCACAGAA	0	
	2/45	**CC**A**C**TAGGAGTCGTAGCACAGGGAGCTGTACAGTCACAGAA	1	1
	2/45	**CC**ATTAGGAGTCGTAGCACAG**A**GAGCTGTACAGTCACAGAA	1	19
	2/45	**CC**ATTAGGAGTCGTAGCACAGGGAGCTGTACAGTCACAGA**G**	1	38
	2/45	**CC**ATTAGGAGTCGTAGCACAGGGAGCTGTACAGTCA**GTCG**A	4	34–37
	2/45	**CC**ATTAGGAGTCGTAGCACAGGGAGCTGTAC**GTCG**AC**CTGC**	8	29–32, 35–38

PAM motifs are in bold type and underlined at the 5′ ends and the positions and identities of mismatches that were introduced are also in bold type and underlined for: (A) genes encoding ATV618, ATV892 and ATV145 and cloned protospacers of *S. solfataricus* P2 CRISPR loci, and (B) cloned protospacers of *S. islandicus* REY15A in CRISPR locus 2.

### CRISPR loci deletions are not dependent on protospacer transcription

Each of the above changes observed in the CRISPR loci will prevent both the CRISPR/Cas and CRISPR/Cmr systems from targeting the cloned viral genes or their transcripts respectively (Marraffini and Sontheimer, 2008; Hale *et al*., 2009; Shah *et al*., 2009). In order to inhibit transcription and remove the possibility of viral gene transcripts feeding back onto the CRISPR loci and inducing spacer deletions, vectors were constructed for the genes of ATV145 and ATV618 where no transcription occurred. Promoter regions were replaced by an elongated archaeal T-rich terminator region (see *Experimental procedures*). For both these constructs, again transformation efficiency was very low and two transformants were analysed from each construct. For the ATV145 construct, CRISPR locus D deletions of 4.4 and 4.6 kb were estimated from PCR reactions (Fig. 3C) and for the ATV618 constructs, CRISPR locus D deletions of 3.3 and 3.4 kb were detected. Subsequent sequencing showed that all four lacked matching spacers 28 or 29 respectively (Table 1). For ATV145, the crRNA is complementary to the reverse transcript and it was established by Northern blottings that there was no transcription on either strand across the protospacer (Fig. 4B). For ATV618, where the crRNA is complementary to the mRNA, using the same vector construct, no transcription was observed in Northern blots (data not shown). This result eliminates the possibility that the deletions occurring within the CRISPR loci resulted from a feedback mechanism involving transcripts from either strand of the gene.

### Some CRISPR/Cas modules may be inactive

CRISPR locus F has been shown, exceptionally, to be identical in sequence between *S. solfataricus* strains P1 and P2, and to lack a leader region and some *cas* genes (Lillestøl *et al*., 2006; 2009;). This suggested that its capacity to add new spacers had been impaired. Therefore, we investigated whether CRISPR locus F was affected by vector-borne matching protospacers. Spacer 67 of CRISPR locus F (Fig. 2) matches imperfectly (three mismatches) the gene of a putative partitioning protein NOB8-315 encoded by the *Sulfolobus* conjugative plasmid pNOB8 (She *et al*., 1998). The gene with three mismatches was inserted into pEXA2 cloning site in addition to a mutated gene with a perfect match. Constructs were also prepared with and without the family I CC PAM motif but each construct produced transformation efficiencies similar to the empty vector [on average 10^4^ cfu (µg DNA)^−1^] and no deletions of spacer 67 were detected in the sequenced PCR products (Table 1). Therefore, we infer that cluster F is inactive and this is compatible with the observation that the RNA transcript from cluster F is incompletely processed such that the active crRNA components, in the size range 35–45 nt, are not formed (Fig. S1).

### Protospacers carrying mismatches also affect CRISPR loci in *Sulfolobus islandicus* REY15A and *S. solfataricus* P2

Given that the CRISPR loci changes were observed independently of whether viral genes carrying matching protospacers were transcribed, we inferred that it was sufficient to perform further experiments with vector-cloned protospacer DNAs carrying appropriate PAM motifs (Lillestøl *et al*., 2009). Moreover, since a reliable and stable genetic system has recently been developed for *Sulfolobus islandicus* REY15A (Deng *et al*., 2009), which also carries a much simpler CRISPR/Cas system with only a tandem pair of CRISPR loci 1 and 2 (and two *cmr* cassettes) (Fig. 2B), we performed a series of parallel experiments in which we synthesized and cloned protospacers matching to selected CRISPR spacers, or combinations of spacers, for both *S. solfataricus* P2 and *S. islandicus* REY15A (Fig. 2).

Several vector constructs were prepared each carrying a protospacer showing increasing degrees of sequence mismatches (listed in Table 2) to spacer 45 of family I CRISPR locus 2 of *S. islandicus* REY15A and to spacer 28 of family II CRISPR locus A of *S. solfataricus* P2 (Fig. 2) and the constructs carried the protospacer PAM motifs CC for the former and CT for the latter (Table 2). Both spacers were 38 bp in length and constructs were prepared with perfect matches, one mismatch at spacer positions 1, 19 or 38, two mismatches exclusively for *S. solfataricus* at positions 1 and 38, and additional constructs were made with four mismatches (positions 34–38) and 8 or 10 mismatches (positions 29–38) all at or near the 3′ end (Table 2). For each construct, including the 8–10 mismatches at the 3′ end, low transformational efficiencies were observed compared with the empty vector (Table 3). Furthermore, for all except the latter constructs, some deletions were observed within CRISPR loci covering the matching spacer (Table 2).

**Table 3:** Deletions observed in CRISPR locus 2 of *S. islandicus* REY15A and locus A of *S. solfataricus* P2.

		Deletions in *S. islandicus* CRISPR locus 2		Deletions or insertions in *S. solfataricus* CRISPR locus A	
			
Experiment	Mismatches in protospacer 45 or 28 (38 bp)	PCR estimate (kb)	Sequencing result	PCR estimate (kb)	Sequencing result
P1	0	64 bp**	r45–r46 (×3)	4.5	r24–r93
		3.7 (×2)	n.d.	3.8*	r7–r64
		2.7*	r3–r47	6.4*	r2–r103
		2.5 (×2)	n.d.	0 (×2)	n.d. (×2)
		2.2	r40–r69		
		1.7 (×2)**	r30–r53		
			r22–r47		
		0.5	r38–r52		
		0 (×5)	n.d. (×5)		
P2	1 (+1)	4.7	n.d.	+1, 6.4	n.d.
		0	n.d.	+1	ISC1359 in sp28
				0, 4.4	n.d.
				6	n.d.
P3	1 (+19)	64 bp	r45–r46	3*	r5–r56
		4.2	n.d.	1	r18–r30
		0	n.d.	+1	ISC1359 in sp28
				0* (×2)	n.d. (×2)
P4	1 (+38)	64 bp	r45–r46	0	Spacer present
		0	n.d.	+1	ISC1359 in sp28 (×2)
P5	2 (+1, +38)	n.d.	n.d.	5.3*	n.d.
				4.8*	r26–r103
				0 (×2)	n.d.
				+1	n.d.
P6	5 (+34−38)	3.4	n.d.	2* (×3)	r5–r52
		1.7	n.d.	1*	r5–r52
		0 (×2)	n.d. (×2)	2	r23–r41
				0	n.d. (×1)
P7	8–10 (+29−38)	0 (×9)	Spacer present (×1)	0* (×5)	Spacer present (×1)

**Fig. 5:**
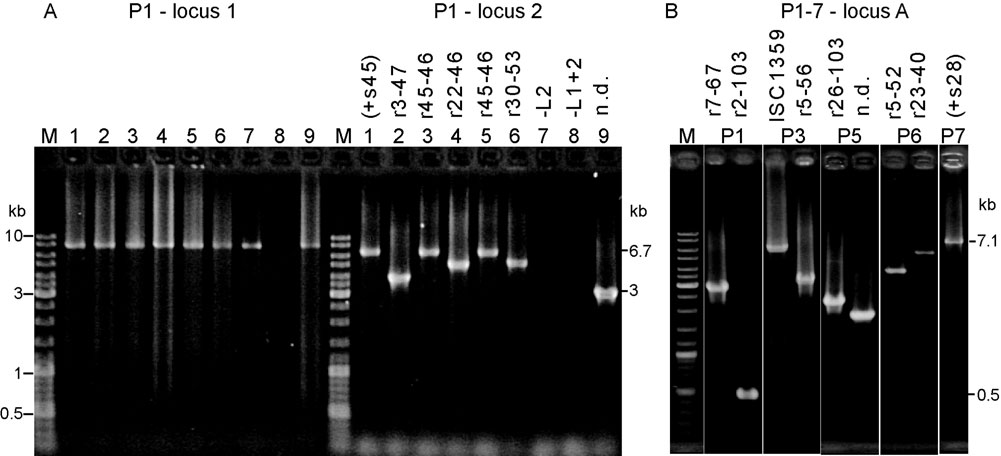
Agarose gels showing examples of PCR products obtained from CRISPR loci of transformants carrying protospacers and CC and CT PAM motifs cloned into pDL1 and pEXA2 respectively. A. *S. islandicus* REY15A where both locus 2 carrying the matching spacer and, as a control, locus 1 were amplified. B. *S. solfataricus* P2. P1 to P7 refer to the experiments listed in Table 3. M indicates DNA size markers. Bracketed spacer numbers denote the presence of the matching spacer. All the illustrated samples carrying deletions are marked with asterisks in Table 3.

CRISPR deletion sizes, or IS element insertions, were estimated from PCR analyses where the results derived from PCR products shown in Fig. 5 are marked by asterisks in columns 3 and 5. ‘0’ means that no deletion was detectable from the PCR product size. ‘Spacer present’ indicates that the CRISPR locus contains the spacer matching the protospacer. n.d. denotes not determined. (×2) shows that the result was obtained independently twice. Transformational efficiencies were low and were estimated at below 3% for the *S. islandicus* REY15A experiments and in the range 0.1–2.0% for *S. solfataricus* P2 (see Table 1 legend and *Experimental procedures*).

PCR products across the deleted regions of locus 2 of *S. islandicus* are shown in Fig. 5A for transformants arising from the perfectly matching protospacers, with control PCR reactions provided for locus 1 showing no changes except for sample 8 where both CRISPR loci had been deleted. These PCR products are indicated in a summary of all the results listed for *S. islandicus* in Table 3. Similarly, for the *S. solfataricus* constructs, examples of PCR products from constructs carrying both perfectly matching, and mismatching, protospacers are given in Fig. 5B, including an example, in sample P3, of an ISC1359 insertion. All the results are summarized in Table 3.

**Fig. 6:**
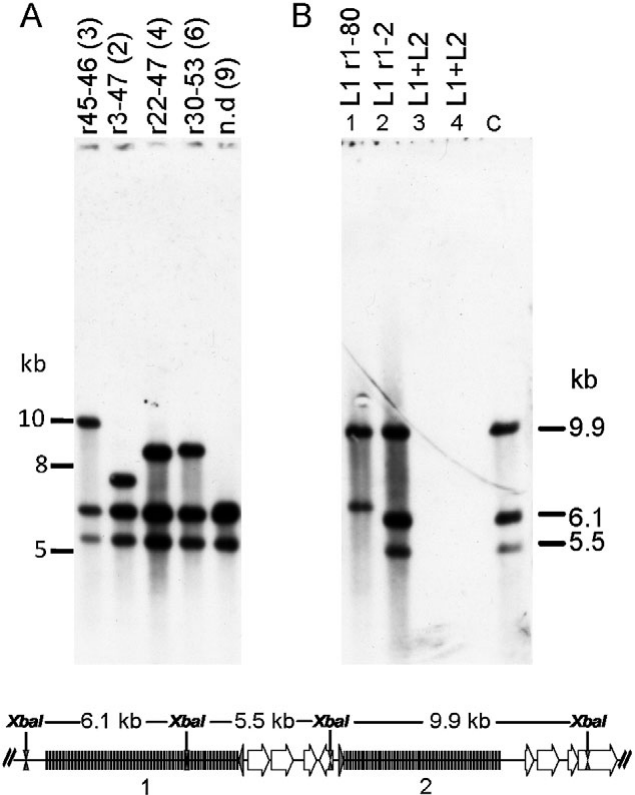
Southern blot analysis of CRISPR locus deletions of *S. islandicus* REY15A transformants where sample C denotes a control sample of *S. islandicus* REY15A. The deleted regions determined by sequencing are indicated above each well. A. Analysis of deletions in locus 2 determined from the PCR products shown Fig. 5A where the numbers in the brackets correspond to the sample numbers on agarose gel. B. Samples correspond to the PCR products illustrated in Fig. 7A and B. Tracks 1 and 2 carry transformants 1 and 2 while tracks 3 and 4 carry transformants with protospacers matching both spacer 20 of locus 1 and spacer 45 of locus 2 (Table 5). The lower diagram shows the restriction sites in the CRISPR region where the expected DNA fragment sizes are indicated.

In a control experiment to confirm that the PCR products reflected the predicted and predominant genomic changes, and not some minor smaller products, Southern blot experiments were performed on the *S. islandicus* CRISPR loci. Results for five of the transformants in Fig. 5A which produced major genomic changes (samples 2, 3, 4, 6 and 9) were tested. The results presented in Fig. 6A all show fragment sizes that are consistent with the sizes of the PCR reactions (Fig. 5A; Table 3)

There was an important difference between the two sets of results. Whereas changes in the CRISPR locus 2 of *S. islandicus* exclusively involved deletions, insertions of the transposable element ISC1359 were observed within matching spacer 28 for some of the *S. solfataricus* transformants, after its target site ACGT (Redder and Garrett, 2006) (Table 3; Fig. 5B). This result is consistent with the relatively high transpositional activity found only in the latter strain (Martusewitsch *et al*., 2000; Redder and Garrett, 2006; Deng *et al*., 2009).

### Testing for the significance of the family I CC PAM motif

A CC PAM motif was predicted to be important for family I crRNA targeting in *Sulfolobus* species, with a low tolerance for a T at either position (Lillestøl *et al*., 2009; Shah *et al*., 2009). Therefore, using the *S. islandicus* genetic system, we prepared a series of constructs with protospacers matching spacer 45 of CRISPR locus 2 (Fig. 2) with variants of the CC motif. High transformation levels (see *Experimental procedures*) were observed with motifs GG, GA and TT, and there was no evidence of deletions in CRISPR loci among the transformants examined (Table 4). The results obtained with CT and TC were less clear. The former yielded relatively high transformation efficiency but produced a single large deletion including the matching spacer, for one of six transformants examined, while the TC motif produced few transformants but no evidence of deletions in the four transformants examined. Only CC yielded low transformation efficiencies and high levels of deletions, for 12 of the 17 transformants examined (Table 3). These experimental results confirm that the predicted CC PAM motif is required for targeting foreign DNA *in vivo* for the family I CRISPR/Cas system of *Sulfolobus* (Lillestøl *et al*., 2009) thereby facilitating the distinction of foreign protospacer sequences from host CRISPR spacer sequences which carry the equivalent motif AA in their repeat.

**Table 4:** Challenging the CRISPR/Cas system of *S. islandicus* REY15A (family I) with a protospacer perfectly matching to spacer 45 of CRISPR locus 2, employing alternative dinucleotide PAM motifs.

		Deletions in *S. islandicus* CRISPR locus 2	
		
Locus/spacer + PAM motif	Transformation efficiency [cfu (µg DNA)^−1^]	PCR estimate (kb)	Sequencing result
None	5.2 × 10^5^	0 (×2)	Spacer present (×1)
L2/45 + GG	4.1 × 10^5^	0 (×2)	Spacer present (×2)
L2/45 + GA	6.3 × 10^4^	0 (×2)	Spacer present (×2)
L2/45 + CC	1.7 × 10^2^	See P1, Table 3	See P1, Table 3
L2/45 + CT	2.9 × 10^3^	6	r11–r91
		0 (×5)	Spacer present (×1)
L2/45 + TC	94	0 (×4)	Spacer present (×2)
L2/45 + TT	7 × 10^4^	0 (×3)	Spacer present (×3)

Each construct was tested at least twice and transformation efficiencies were averaged and are approximate (see *Experimental procedures*). (×2) shows that the result was obtained independently twice.

### Mechanism of formation of deletions within CRISPR loci – spontaneous or induced?

The large variety of CRISPR loci deletion mutants produced in these experiments raised the question as to whether they resulted from a low level of spontaneous intracellular recombination or whether the effects are somehow induced. In order to examine this further we performed two additional experiments in the *S. islandicus* system. In the first, a construct was generated carrying two protospacer sequences, matching spacer 20 in CRISPR locus 1 and spacer 45 in CRISPR locus 2 (Fig. 2B), each with an associated CC PAM motif. It was inferred that simultaneous and independent loss of both spacers would be a highly improbable event. Of the very few transformants obtained none generated PCR products from CRISPR loci 1 or 2, suggesting that both were absent (Table 5). This was then confirmed by a Southern blot analysis which demonstrated that both CRISPR/Cas modules 1 and 2 had been deleted. There was no evidence for independent deletions having occurred in the two CRISPR loci (samples 3 and 4 in Fig. 6B).

**Table 5:** Characterization of the CRISPR loci of *S. islandicus* REY15A (family I) after challenging with constructs carrying protospacers matching spacers within locus 1 and/or 2 (Fig. 2).

			CRISPR locus deletion	
			
Experiment	CRISPR locus/spacer (+CC motif)	Transformation efficiency [cfu (µg DNA)^−1^]	PCR estimate (kb)	Sequencing result
1	L2/45 + L1/20	4	> 17 kb (3×)	L1 + L2 (×2)
2	L1/1	6	64 bp (2×)	r1–r2 (×2)
			0.2 and 5	Leader–r3 and r1–r80

Each construct was tested twice and transformation efficiencies were averaged. L1 + L2 indicates that both CRISPR loci are deleted, and r1–r2 shows that recombination had occurred between repeats 1 and 2 with the resultant deletion of spacer 1 and one repeat.

**Fig. 7:**
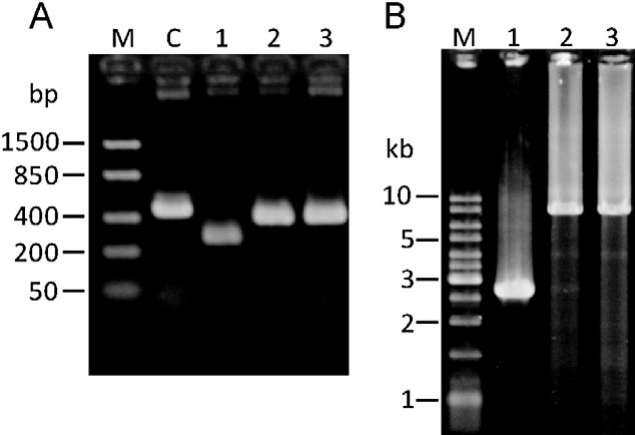
PCR products showing deletions in CRISPR locus 1 produced by a protospacer matching spacer 1 of locus 1 (Table 5).A. PCR from three transformants using the primers bordering spacer 1 of locus 1 where C denotes a control sample run with *S. islandicus* REY15A. B. PCR products obtained from the same transformants (1–3) with primers amplifying the whole of locus 1.

In the second experiment a protospacer matching spacer 1 of CRISPR locus 1 was tested. This has a very low probability of being randomly deleted since deletion as a result of recombination can only occur via repeat 1 adjacent to the leader region. Only three transformants were obtained in the experiment, two of which had specifically lost spacer 1 and a flanking repeat (r1 to r2), and a third that showed a mixture of two types of deletion, one from r1 to r80, the other from within the leader region to r3, both including spacer 1 (Fig. 7A and B; Table 5). Deletion mutants r1–r2 and r1–r80 were examined further by a Southern blot analysis and the sizes of the restriction fragments (samples 1 and 2 in Fig. 6B) correlate with those of the PCR reactions except that only the larger deletion product was detectable for sample 2 (5.5 kb) consistent with it being the dominant deletion in the transformant (Figs 6B and 7).

Next, we investigated whether we could find any experimental evidence for deletions occurring in CRISPR loci of the untransformed *S. solfataricus* P2. PCR products were generated from within CRISPR loci A and D, and a continuum of minor bands were observed, all smaller than the main expected product (Fig. S2). The yields of the smaller products were enhanced by using shorter elongation times in the PCR reactions and a limited size range of these products were cloned and sequenced as indicated, for loci A and D, and the results showed that they carried a range of CRISPR locus deletions with perfectly maintained repeat–spacer junctions (Fig. S2). This indicates that a low level of random CRISPR loci deletion variants is produced during amplification. Moreover, the presence of perfectly maintained repeat–spacer boundaries in all the sequenced variants (Fig. S2) suggests that deletions occur as a result of homologous recombination between the repeats.

This last result has important implications for how the CRISPR loci deletions are generated *in vivo* (see *Discussion*) but they also helped to resolve a major practical problem that recurred throughout this study. For the experiments summarized in Table 3, PCR products from about 80 individual transformants were sequenced for each *Sulfolobus* strain. Despite the appearance of strong homogeneous PCR products, carrying deletions, on agarose gels, about 30 products for each strain yielded mixed sequence traces (which include the samples indicated by n.d. in Fig. 5). We infer the latter were caused by sequence heterogeneity generated by recombination resulting in the random loss of a few spacers during the PCR reactions. Many of these PCR products were subsequently cloned and resequenced successfully.

## Discussion

The experiments mimic, to a degree, the chronic invasion of *Sulfolobus* cells by viruses, conjugative plasmids or other aggressive genetic elements. They differ in that when the protospacer-carrying vectors are targeted and degraded by the host CRISPR immune systems this should lead to cell death due to lack of uracil synthesis. The survival of very few transformants, for most of the constructs tested which carried perfectly or closely matching protospacers, confirms that the majority of cells do indeed die. Moreover, most, but not all of the cultured transformants survived as a result of either deletions occurring within the chromosomal CRISPR locus, including the matching spacer, or as a result of deletion of whole CRISPR/Cas modules.

**Fig. 8:**
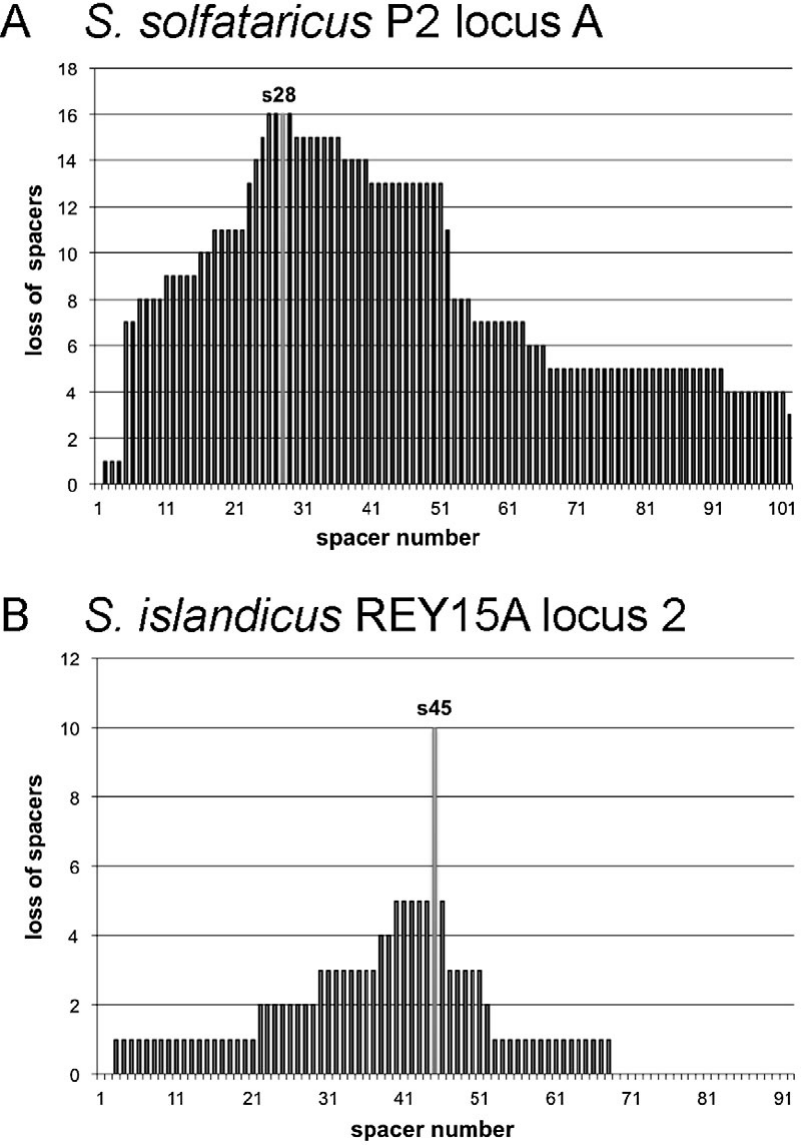
Distributions of deletions within CRISPR loci produced by constructs carrying (A) ATV viral genes or protospacer sequences matching spacer 28 of CRISPR locus A of *S. solfataricus* P2 and (B) protospacer sequences matching spacer 45 of CRISPR locus 2 of *S. islandicus* REY15A.

The mechanism by which deletions occur within CRISPR loci is puzzling, and this is reinforced by a summary of the sequencing data for CRISPR locus A of *S. solfataricus* and locus 2 of *S. islandicus* (Tables 1 and 3) and in histograms (Fig. 8). The numerous sequenced deletion variants reveal a wide range of deletion sizes with very few being identical. Moreover, although the two distributions are qualitatively similar with broad distributions peaking at the matching spacer, there are significant differences. On average, the deletions in *S. solfataricus* are larger and in *S. islandicus*, there is a high incidence of specific deletions of single matching spacers (and an adjacent repeat) that are not observed for *S. solfataricus* (Fig. 8A and B). The latter effect was also reinforced by the results of the experiment (Fig. 7; Table 5) where two of the three analysed *S. islandicus* transformants had specifically lost the matching spacer 1 of CRISPR locus 1. Not included in the histograms is the selective targeting of the matching spacer by ISC1359 in *S. solfataricus* (Table 3; Fig. 5B).

The data for both *Sulfolobus* species (Fig. 8), and the results for the experiments targeting spacer 1 of CRISPR loci 1 in *S. islandicus* (Table 5), are compatible with the occurrence of a low level of spontaneous recombination activity occurring within CRISPR loci, intracellularly, either during replication or during the G_2_ phase of the cell cycle when two chromosomal copies are present per cell for longer periods (Bernander, 2007). This could result in the formation of viable transformants carrying vector-borne protospacers in cells that have undergone deletion of matching CRISPR spacers. This hypothesis is also consistent with the low level of random recombination events occurring between repeats observed during PCR reactions performed on whole, and different, CRISPR loci (Fig. S2). It is also supported by the amplification of two distinct deletion products from the same transformant as shown for two of the four P2 samples in Table 3, and also for the samples 1 in Fig. 7A and B where deletions of 0.2 kb and 5 kb were observed for one transformant albeit with the larger product dominating (Table 5; Fig. 6B).

This suggests that recombination can occur intracellularly during colony development or culturing. However, the data for *S. islandicus* (Fig. 8B) indicate that there may be an alternative mechanism operating, given the frequency of specific spacer deletions that are observed (50% in Fig. 8B). It raises the possibility that the spacer deletions are somehow induced in *S. islandicus* either by the matching vector-borne protospacer within the construct, or by the host itself, since we have eliminated the possibility experimentally that transcripts carrying the protospacer sequence could have a feedback role (Table 1; Fig. 3C). There remains the possibility that a very low level of crRNA feedback and targeting of the matching chromosomal spacer can occur. Subsequent recombination at the repeats bordering the cut spacer might then be the most effective mechanism for chromosomal repair.

The operation of the spontaneous but precise repeat-based recombination mechanism could also explain how the CRISPR loci can change relatively quickly in natural environments including the multiple indels, probably deletions, observed in the large corresponding CRISPR loci of three sequenced *S. solfataricus* strains P1, P2 and 98/2 (Lillestøl *et al*., 2006; 2009; Shah and Garrett, 2010), but also the more extreme case of the complete lack of shared CRISPR loci spacer regions in sequences of several very closely related *S. islandicus* strains isolated from a few localized geographical locations (Held and Whitaker, 2009; Shah *et al*., 2009; L. Guo *et al*., submitted).

Clearly, *Sulfolobus* cells adapt to being challenged by protospacers that match spacers in their active CRISPR loci primarily by losing the matching spacer. However, a significant proportion of the viable transformants (about 20%) retained their perfectly matching spacers to ATV genes (Table 1). Moreover, searching for evidence of mutations in the *cas* gene cassettes or leader regions including the CRISPR loci promoters of a few of the viable *S. islandicus* transformants failed to reveal any changes, although such defects which occur naturally in the *cas* gene cassettes and leader region appear to explain the inactivity of CRISPR locus F of *S. solfataricus* (Table 1) (Lillestøl *et al*., 2009). This implies that there has to be a mechanism, *in trans*, for shutting down CRISPR transcription and/or processing, possibly involving the post-translational modification of a Cas protein. A possible effector could also be the *Sulfolobus* repeat-binding protein (Peng *et al*., 2003) which can alter transcription from CRISPR loci (L. Deng, unpublished) but there may be other silencing systems including inhibiting the CRISPR locus promoter within the leader region, as have been observed in a bacterium (Pul *et al*., 2010). We plan to sequence one or more genomes of the *S. islandicus* transformants in an attempt to gain more insight into this process.

Finally, the results serve to emphasize further the diversity of the CRISPR/Cas and CRISPR/Cmr systems, and the differences occurring between, and among, archaeal and bacterial systems. Thus, the target discrimination mechanism of the crRNA–Cas protein complex differs from that of the bacterium *Staphylococcus epidermidis* which specifically targets complementary protospacers on foreign DNA when any sequence mismatches occur between the short repeat sequence at the 5′ end of the crRNA (Marraffini and Sontheimer, 2010). Here we show that matching to the *Sulfolobus* family I CC PAM motif is important for crRNA to target foreign DNA, with other mismatching motifs GA, GG and TT having no effect. Moreover, whereas the *S. thermophilus* CRISPR/Cas apparatus requires perfectly matching spacer–protospacer sequences to be activated (Barrangou *et al*., 2007), the *Sulfolobus* system appears to be much less stringent. One possible explanation to reconcile all these differences is that the PAM motif interaction compensates for the imperfect annealing of crRNA to the protospacer in the *Sulfolobus* systems. This apparent flexibility of the targeting mechanism may also reflect the much wider variety and diversity of viruses and plasmids which inhabit these hyperthermophilic crenarchaea (Prangishvili *et al*., 2006a).

## Experimental procedures

### Growth of *Sulfolobus* cells and general DNA manipulations

All *Sulfolobus* cells were grown at 75–78°C in complex medium TYS or selection medium SCV and competent cells were prepared and transformed as described (Deng *et al*., 2009). Arabinose was added to media to a final concentration of 0.2% to activate the *araS* promoter (the promoter for the arabinose-binding protein) of pEXA2 (Deng *et al*., 2009).

Standard methods of cloning and other DNA manipulations were used (Sambrook and Russell, 2001). DNA restriction and modification enzymes were purchased from New England Biolabs (Hitchin, UK) or Fermentas (St. Leon-Rot, Germany), Ex *Taq* DNA polymerase was purchased from Takara (Otsu, Japan) and *Pfu* polymerase was obtained from Fermentas (St. Leon-Rot, Germany). Total DNA was isolated using DNeasy Kit (Qiagen, Westberg, Germany). Plasmid DNA was isolated from *E. coli* or *Sulfolobus* cells using QIAprep Spin Miniprep kit (Qiagen Westberg, Germany) or NucleoSpin Plasmid Kit (Macherey-Nagel, Düren, Germany). PCR products were purified by QIAquick PCR purification kit (Qiagen, Westberg, Germany). All primers were synthesized by TAG Copenhagen A/S (Copenhagen, Denmark). DNA size marker used were: O'GeneRuler™ DNA Ladder Mix, 100–10 000 bp and FastRuler™ DNA Ladder, 50–1500 bp (Fermentas).

### Strains and vector construction

The *S. solfataricus* P2 vector host *S. solfataricus* P2-InF1 carried a *pyrF* gene inactivated by a single copy of ISC1225 and the *S. solfataricus* vector carried *Sulfolobus acidocaldarius pyrE/F* genes. For *S. islandicus*, the double-deletion mutant *S. islandicus* E233S was used, which carried a large *pyrEF* gene deletion and a complete *lacS* gene deletion (Deng *et al*., 2009).

pDL1 was constructed by self-ligation of the PvuII- and ZraI*-*digested pZC1 large fragment (Peng *et al*., 2009). pEXA was produced by the triple ligation of products of: SphI- and AvaI-digested pDL1; SphI- and NdeI-digested *araS* promoter fragment, and NdeI- and SphI-digested multiple cloning site (MCS) fragment. The *araS* promoter fragment was amplified by primer araSF (5′-gcGCATGCTTTTTTTTAGAAAAACATCCAATATGTTAAC-3′) and araSR (5′-ttttttttCATATGCTCGGGTACTTTTATGACCTAAC-3′) using total *S. solfataricus* P2 DNA as template (underlined nucleotides indicate restriction digestion sites and small letters constitute protection nucleotides for restriction sites).

The MCS fragment was synthesized by primers MCS1, MCS2, MCS3, MCS4, MCS5 and MCS6 (MCS1-5′ ttttttttCATATGCATCATCACCATCATCATAGTAGTGGTTTAGT; MCS2-5′CATCGATTCGCGATCCCCTTGGTACTAAACCACTACTATGATGATGGTGA; MCS3-5′AAGGGGATCGCGAATCGATGCTAGCTACGCGTCTCCGGATGTACAAAGGC; MCS4-5′ATCAGCGTCGACCTTATCGTCATCATCAGGCCTTTGTACATCCGGAGACG; MCS5-5′GACGATAAGGTCGACGCTGATCAAGCGGCCGCACACCATCATCATCACCA; MCS6-5′aaCCCGGGAAAAAAAAGATTTTGCTTAGTGGTGATGATGATGGTGTGCG) as follows: all primers were added to the final concentration of 0.2 µM in a standard 50 µl PCR reaction: 94°C for 20 s, 65°C for 20 s and 72°C for 20 s over 15 cycles. One microlitre of the product was then added as DNA template to a standard PCR reaction using MCS1 and MCS6 as primers. After 20 cycles of 94°C for 20 s, 65°C for 20 s and 72°C for 20 s, the MCS fragment was synthesized.

To construct protospacer-containing vectors, SOE-PCR was performed to generate protospacer fragments. Briefly, primers were designed with about 18 nt overlap at their 3′ ends and were added to a standard PCR reaction, run at 95°C for 3 min, 30 cycles at 95°C for 15 s, 40°C for 15 s and 68°C for 5 s. The product was then purified by a Nucleotide removal kit (Qiagen, Westburg, Germany), digested overnight and repurified using this kit. Ligation was performed by T4 ligase with the corresponding digested vector at 22°C for 1 h. The ligation product was transformed into *E. coli* DH5α directly by heat shock following a standard protocol (Sambrook and Russell, 2001) and transformants were screened by colony PCR and verified by sequencing.

For *S. solfataricus* experiments, the *S. solfataricus pyrEF* genes in pEXA were replaced by *pyrEF* genes from *S. acidocaldarius* to minimize the possibility of homologous recombination. The pEXA vector was fully digested with Cfr91, destroying one of three KpnI sites on the vector and then partially digested with KpnI to remove the *pyrEF* genes and the cut vector was purified with a QIAquick gel extraction kit (Qiagen). The *S. acidocaldarius pyrEF* genes were amplified with primers S.aci1: 5′-tcccCCCGGGAG**A**AAA**A**AAAGCTATGGATATTGTCTTACCAC-3′ and S.aci2: 5′-cgg GGTACCag**a**aa**aa**aaatctgttgtgggaacttcac-3′, purified and cut with Cfr91 and KpnI and then inserted into the linear pEXA to produce pEXA2. Bold nucleotides represent mismatches to the genomic sequence to enable transcriptional terminators to be inserted into the vector on both sides of the *pyrEF* genes. The integrity of the whole construct was confirmed by DNA sequencing.

To remove the *araS* promoter from pEXA2, the plasmid was digested with SphI and NdeI. To inhibit transcription, a terminator sequence was generated using the primer set Term/SphI: 5′-acatGCATGCTTCTTTTTCTTTCCCTTCTTTTTTTACC-3′ and Term/NdeI: 5′-ggaattcCATATGAAAAAGAGGTAAAAAAAGAAGGG-3′ and inserted into the cut vector.

### Gene constructs

pEXA2 was cut with MluI and EagI and purified with QIAquick gel extraction kit (Qiagen, Westberg, Germany). Primers with overhanging restriction sites were used to amplify the genes from an in-house libraries of the ATV virus and pNOB8. PCR fragments were purified using either QIAquick PCR purification kit or QIAquick gel extraction kit (Qiagen, Westberg, Germany), before cutting with MluI and EagI and ligating into the cut pEXA2 vector. Correct inserts were verified by restriction analyses and DNA sequencing.

Site-directed mutagenesis was accomplished using mutagenic primers as described by Heckman and Pease (2007). Internal mutagenic primers are designed overlapping one another and used with the original gene construct primers to create two intermediate PCR products. These PCR products were then used as template DNA for a second round of PCR to generate the mutated gene.

### Determination of transformation efficiencies

Transformation efficiency measurements for the two *Sulfolobus* strains used are not quantitative for two main reasons. First, the plating material Gel-rite generally contains low and variable levels of pyrimidine compounds, some of which survive autoclaving, and therefore some non-transformants always appear on plates. Moreover, as discussed earlier for the two-layer Gel-rite plating system, some non-transformants appear on plates because of pyrimidines liberated from lysis of dead cells during the long (7–21 days) incubations (Deng *et al*., 2009). We invariably distinguished transformants from non-transformants by growing them in uracil-deficient media after plating, and for all the experiments where CRISPR loci underwent deletions there were very few viable transformants. Experiments included in Tables 1 and 3 were performed over a 3-year period using different batches of Gel-rite, and competent cells, and therefore we only give the approximate low percentage range of the control transformation efficiencies. Experiments involving altering the PAM motif (Tables 4 and 5) were performed over a shorter time period with one batch of Gel-rite and competent cells. The transformation efficiencies given are averaged and approximate.

### Localizing CRISPR locus deletions

PCR reactions were performed with either *Taq* or *Pfu* polymerase. Annealing temperatures used were 5°C lower than the estimated Tm of the primer. Elongation times allowed for about 1 min per kilobase pair of the expected product. For the experiment in Fig. S2 elongation times were reduced from 7 min to 2.5 min to enhance the yields of the smaller products. Amplified DNA was purified and generally ran on 0.8% agarose gels with DNA size markers (GeneRuler™ DNA Ladder Mix, 0.1–10 kb, Fermentas).

PCR products were obtained across the chromosomal regions of *S. solfataricus* P2 CRISPR loci A to D and of *S. islandicus* REY15A CRISPR loci 1 and 2 using pre-mixed Ex *Taq* according to the manufacturer's protocol with 75–100 ng of genome DNA in a 10 µl reaction. For *S. solfataricus*, primer pairs AF: 5′-TGGCGGTTATTAATTGGGA-3′ and AR: 5′-TTGCGGATTCTTGACGTG-3′ and DF: 5′-GCACGCTTCCTACCTTCATTTCCACTAC-3′ and DR: 5′-CATCCCTCTAACCCTTCCCAACCTCATA-3′ were used to amplify the CRISPR loci A and D respectively. For *S. islandicus* C1F: 5′-AGCTTGCTTACCTCAAGGTACTTTACGT-3′ and C1R: 5′-TTAATAAACGACGATTTTCCTCTTGAT-3′, C2F: 5′-AGGATAGCGAAGTCGTAG AGTTTGGAT-3′ and C2R: 5′-TAACGCACGGTATTGAAACTTCTCATC-3′ were used to amplify CRISPR loci 1 and 2 respectively. Purified PCR products were sequenced directly (Eurofins MWG Operon, Ebersberg, Germany) or first cloned using CloneJET™ PCR Cloning Kit (Fermentas, St. Leon-Rot, Germany) and then sequenced.

### RNA preparation and Northern blotting

Total RNA was prepared using Trizol (Invitrogen, Paisley, UK) according to the Invitrogen protocol essentially as used for extracting plant si-RNAs (Sunkar *et al*., 2005). For Northern blotting of small RNAs, 20 µg of RNA was mixed with 10 µl of Gel Loading Buffer II (Applied Biosystems/Ambion, Austin, USA) and fractionated in a 6–10% polyacrylamide gel containing 7 M urea, 90 mM Tris, 90 mM boric acid, 2 mM EDTA, pH 8.3, together with a 10–150 nt ladder (Decade Marker System, Ambion, Huntingdon, UK) or a 0.1–2.0 kb RNA ladder (Invitrogen). Procedures for transferring, and immobilizing RNA on nylon membranes, pre-hybridizing, end-labelling of complementary nucleotides, hybridization and film exposure followed the protocol described earlier (Lillestøl *et al*., 2009). The following oligonucleotides probes were used for Northern blotting: 5′-TCGGTTCCACATCTAGTTATCAAA-3′ to detect transcription from spacer 29 of CRISPR locus D of *S. solfataricus* P2; 5′-CCAATCCCAAGATACATCATCG-3′ to detect reverse transcription from the ATV145 gene, and 5′-CGATGATGTATCTTGGGATTGG-3′ to determine forward transcription from ATV145 gene. The latter unlabelled probe was also used as positive control sample for the detection of ATV145 reverse transcription.

### Southern blotting

Southern hybridizations followed a standard procedure (Sambrook and Russell, 2001). Genomic DNA was prepared and about 2 µg of total DNA of each sample was digested with XbaI. Resulting DNA fragments were fractionated by agarose gel electrophoresis on a 0.8% agarose gel and transferred onto an IMMOBILON-NY+ membrane (Millipore, Billerica, USA) via capillary transfer. DNAs on the membrane were then auto-cross-linked by the UV Cross-linker (Stratagene, La Jolla, USA). Hybridization probes were amplified by primer probes F 5′-GAAAGTCGCTATTGTCAAAG-3′ and R 5′-AGTCTCATACCCGTTCTCAAAG-3′, purified and labelled with Digoxigenin Labelling kit (Roche, Mannheim, Germany). Hybridization was performed at 59°C overnight. The hybridization signals were detected using the DIG detection kit with the CDP-Star (Roche) with the results recorded by exposing the membrane to CL-XPosure™ X-ray films (Thermo Scientific, Rockford, USA).
