# Supplementary material for: Dynamic properties of the Sulfolobus CRISPR/Cas and CRISPR/Cmr systems when challenged with vector-borne viral and plasmid genes and protospacers
Source: Mol Microbiol. 2011 Jan;79(1):35–49. doi: 10.1111/j.1365-2958.2010.07452.x (PMC3025118; doi:10.1111/j.1365-2958.2010.07452.x)
Supplement: Supplementary file 1 [file mmi0079-0035-SD1.pdf]

Figure S1. Northern blot analysis of the processed RNA generated from CRISPR locus F from total cellular RNA extracts of *S. solfataricus* P2. Both 1. spacer 11 of locus F, and 2. the repeat sequence of cluster F (and E) were probed with matching oligonucleotide. M1 and M2 are DNA size ladders.

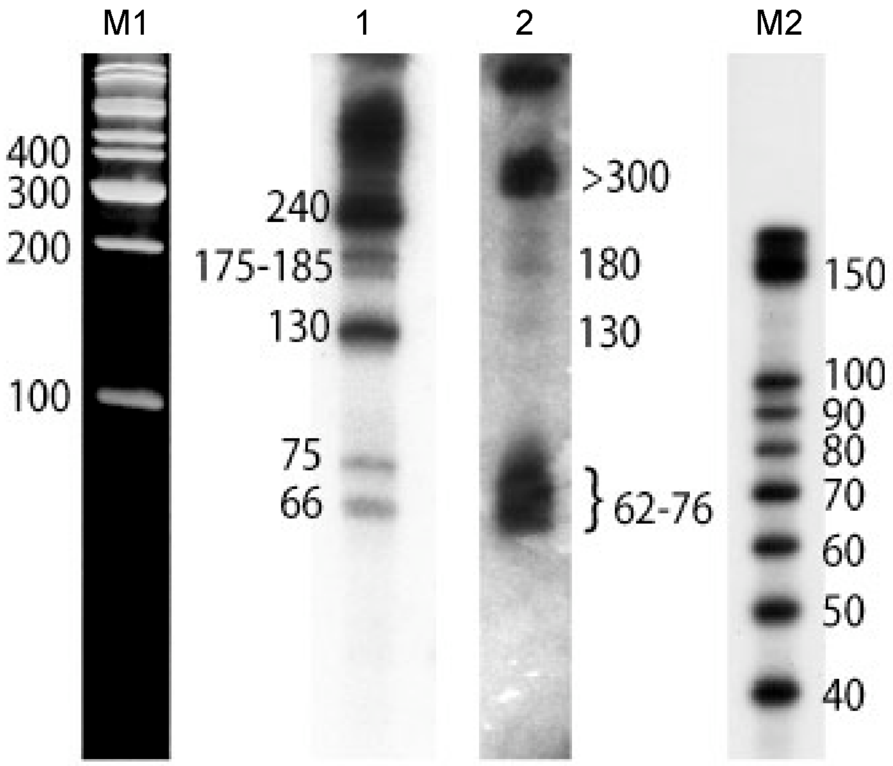

Figure S2. PCR amplification of CRISPR loci A and D of *S. solfataricus* P2. A. Agarose gel showing the position of the main 7.2 kb fragment amplified from locus A where the bracketed region of the gel shows the continuum of fragments that were cloned and sequenced. Shorter PCR elongation times (2.5 min. instead of 7 min.) were used to incease the relative yields of the smaller fragments. B. A summary of the sequencing results indicating the numbers of deleted spacers within locus A. The sequencing results from a closely similar experiment where a 7.1 kb fragment was amplified from locus D are also presented, again showing the deletion sizes detected for a small size range of DNA fragments. Each of the products had recombined at repeat sequences generating perfect repeat-spacer junctions.

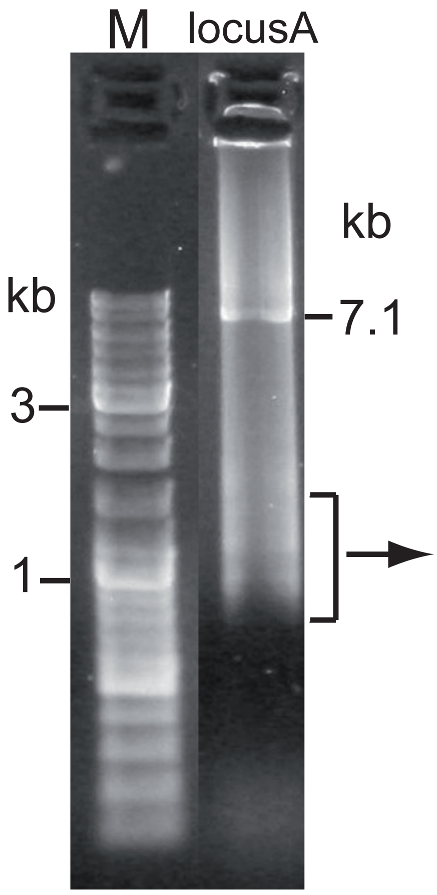

CRISPR locus A		CRISPR locus D	
**deleted spacers**	**sequenced clones**	**deleted spacers**	**sequenced clones**
1-85	3	1-71	1
1-88	1	1-88	1
1-100	2	1-95	5
2-90	1	2-95	1
2-96	1	4-92	1
3-101	1		
3-102	2		
6-101	2		
6-102	2		
8-102	1		
11-101	1		
11-102	3		
